# Extension of Murray's law using a non-Newtonian model of blood flow

**DOI:** 10.1186/1742-4682-6-7

**Published:** 2009-05-15

**Authors:** Rémi Revellin, François Rousset, David Baud, Jocelyn Bonjour

**Affiliations:** 1 Université de Lyon, CNRS, INSA-Lyon, CETHIL, UMR5008, F-69621, Villeurbanne, France, Université Lyon 1, F-69622, France; 2 Department of Obstetrics and Gynaecology, University Hospital of Lausanne, Maternity-CHUV, CH-1011 Lausanne, Switzerland

## Abstract

**Background:**

So far, none of the existing methods on Murray's law deal with the non-Newtonian behavior of blood flow although the non-Newtonian approach for blood flow modelling looks more accurate.

**Modeling:**

In the present paper, Murray's law which is applicable to an arterial bifurcation, is generalized to a non-Newtonian blood flow model (power-law model). When the vessel size reaches the capillary limitation, blood can be modeled using a non-Newtonian constitutive equation. It is assumed two different constraints in addition to the pumping power: the volume constraint or the surface constraint (related to the internal surface of the vessel). For a seek of generality, the relationships are given for an arbitrary number of daughter vessels. It is shown that for a cost function including the volume constraint, classical Murray's law remains valid (i.e. Σ*R*^*c *^= *cste *with *c *= 3 is verified and is independent of *n*, the dimensionless index in the viscosity equation; *R *being the radius of the vessel). On the contrary, for a cost function including the surface constraint, different values of *c *may be calculated depending on the value of *n*.

**Results:**

We find that *c *varies for blood from 2.42 to 3 depending on the constraint and the fluid properties. For the Newtonian model, the surface constraint leads to *c *= 2.5. The cost function (based on the surface constraint) can be related to entropy generation, by dividing it by the temperature.

**Conclusion:**

It is demonstrated that the entropy generated in all the daughter vessels is greater than the entropy generated in the parent vessel. Furthermore, it is shown that the difference of entropy generation between the parent and daughter vessels is smaller for a non-Newtonian fluid than for a Newtonian fluid.

## Introduction

Since several decades, many studies have been carried out on the optimal branching pattern of a vascular system. Based on the simple assumption of a steady Poiseuille blood flow, the well known Murray's law [1] has been established. It links the radius of a parent vessel *R*_0 _(immediately upstream from a vessel bifurcation) to the radii of the daughter vessels *R*_1 _and *R*_2 _(immediately downstream after a vessel bifurcation) as *R*_0_/*R*_1 _= *R*_0_/*R*_2 _= 2^-1/3^. From Murray's analysis, the required condition of minimum power occurs when *Q *∝ *R*^3 ^where *Q *denotes the volumetric flow. This relation, called "cube law", is determined assuming that two energy terms contribute to the cost of maintaining blood flow in any section of any vessel: (i) the pumping power and (ii) the energy metabolically required to maintain the volume of blood which is referred to as "volume constraint". A generalization of this relation can be proposed as *Q *∝ *R*^*c *^where *c *is determined from the condition of minimum power by assuming other constraints (for instance surface constraint yields *Q *∝ *R*^2.5 ^[2]). Under the condition *c *= 3, the shear stress on the vessel walls is uniform and independent of vessel diameter [3]. Several studies have been carried out to determine the value of *c *[4-8] which usually ranges between 2 and 3. The influence of the value of *c *from 2 to 4 has also been investigated [9]. The in vivo wall shear stress in an arterial system has been measured [10]. It was found that mean wall shear stress was far from constant along the arterial tree, which implied that Murray's cube law on flow diameter relations could not be applied to the whole arterial system. According to the authors, *c *likely varies along the arterial system, probably from 2 in large arteries near the heart to 3 in arterioles. A method allowing for estimation of wall shear rate in arteries using the flow waveforms has been developed [11]. This work allowed to determine the time-dependent wall shear rates occurring in fully developed pulsatile flow using Womersley's theory. They found a non-uniform distribution of wall shear rates throughout the arterial system.

Following the cubic law, Murray [12] proposed the optimal branching angle. Optimally, the larger branch makes a smaller branching angle than the smaller branch. This work was extended to non-symmetrical bifurcations [13]. The arterial bifurcations in the cardiovascular system of a rat have been investigated [14]. The results were found to be consistent with those previously reported in humans and monkeys. Murray's optimization problem has also been reproduced computationally using a three dimensional vessel geometry and a time-dependent solution of the Navier-Stokes equations [15].

From Murray's law, some relationships have been proposed between the vessel radius and the volumetric flow, the average linear velocity flow, the velocity profile, the vessel-wall shear stress, the Reynolds number and the pressure gradient [9]. In the same way, based on the Poiseuille assumptions, scaling relationships have been described between vascular length and volume of coronary arterial tree, diameter and length of coronary vessel branches and lumen diameter and blood flow rate in each vessel branch [16,17].

It is also possible to determine Murray's law using other approaches. A model have been suggested based on a "delivering" artery system of an organ characterized, (i) by the space-filling fractal embedding into the tissue and (ii) by the uniform distribution of the blood pressure drop over the artery system [18]. The minimalist principles were not used but the result remains the same. Murray's energy cost minimization have been extended to the pulsatile arterial system, by analysing a model of pulsatile flow in an elastic tube [19]. It is found that for medium and small arteries with pulsatile flow, Murray's energy minimization leads to Murray's Law.

Surprisingly, so far, none of the existing methods on Murray's law deal with the non-Newtonian behavior of blood flow although, the non-Newtonian approach for blood flow modeling looks more accurate. Blood is a multi-component mixture with complex rheological characteristics. Experimental investigations showed that blood exhibits non-Newtonian properties such as shear-thinning, viscoelasticity, thixotropy and yield stress [20-22]. Blood rheology has been shown to be related to its microscopic structures (e.g. aggregation, deformation and alignment of red blood cells). The non-Newtonian steady flow in a carotid bifurcation model have been investigated [23,24]. The authors showed that in that case, viscoelastic properties may be ignored. The fact that blood exhibits a viscosity that decreases with increasing rate of deformation (shear-thining or so called pseudoplastic behavior) is thus the predominant non-Newtonian effect. There are several inelastic models in the literature to account for the non-Newtonian behavior of blood [25,26]. The most popular models are the power-law [27,28], the Casson [29] and the Carreau [30] fluids. The power-law model is the most frequently used as it provides analytical results for many flow situations. On the usual log-log coordinates, this model results in a linear relation between the viscosity and the shear rate. Blood viscosity have been measured by using a falling-ball viscometer and a cone-plate viscometer for shear rate from 0.1 to 400 s^-1 ^[31]. For both techniques, the authors found that measured values are aligned on a straight line suggesting that the power-law model fits experimental data with sufficient precision.

From the literature review, it can be established that none of the existing studies deal with the minimalist principle along with non-Newtonian models. The combination of both aspects will be studied and are presented hereafter. For a seek of generality, the relationships will be given for an arbitrary number of daughter vessels.

### Non-Newtonian model of blood flow

**Figure 1:**
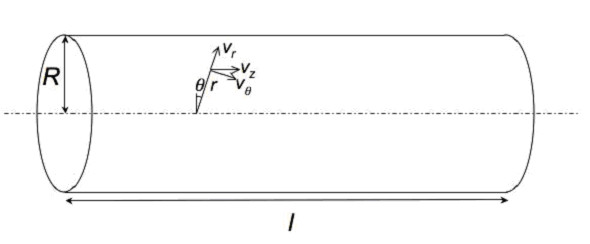
**Definition sketch**.

Consider the laminar and isothermal flow of an incompressible inelastic fluid in a straight rigid circular tube of radius *R *and length *l *as shown in Fig. (1). For steady fully-developed flow, we make the following hypotheses on the velocity components:

(1)

where we have used standard cylindrical coordinates such that the *z*-axis is aligned with the pipe centerline. It means that the only nonzero velocity component is the axial component which is a function of the distance to the pipe centerline only.

The equation of motion may be written as



where *p *denotes the pressure and *σ*_*rz *_is the only nonzero deviatoric stress tensor component. It follows that the pressure is independent from both *r *and *θ*. Moreover ∂*p*/∂*z *is a constant which we will denote as -Δ*p*/*l *identified as the pressure drop over the length *l*. Integrating the third component of the equation of motion, we have:



where *K *is a constant. As the stress remains finite at *r *= 0, the constant *K *must be set to 0. We thus obtain:

(2)

where *σ*_*w *_is the shear stress evaluated at the wall. For a purely viscous fluid, the shear stress *σ*_*rz *_reads:

(3)

where  is the generalized viscosity and  is the effective deformation rate which is given here by .

Let us consider the power-law constitutive equation proposed by Ostwald [27] and De Waele [28] given by:

(4)

It features two parameters: dimensionless flow index n and consistency m with units Pa.s^n^. On log-log coordinates, this model results in a linear relation between viscosity and shear rate. The fluid is shear-thinning like blood (i.e. viscosity decreases as shear rate increases) if n<1 and shear-thickening (i.e. viscosity increases as shear rate increases) if *n *> 1. When *n *= 1 the Newtonian fluid is recovered and in that case parameter m represents the constant viscosity of the fluid. This model is very popular in engineering work because a wide variety of flow problems have been solved analytically for it.

Combining Eqs. (2), (3) and (4) and using the condition of no slip at the wall, we obtain the following velocity field:

(5)

It can be noted that the Poiseuille parabolic velocity profile is recovered for *n *= 1. For a shear-thinning fluid, the velocity profile becomes blunter as *n *decreases. The flow rate is:

(6)

The pressure drop for the flow can be evaluated from Eqs. (2) and (6) to be:

(7)

The previous relation can be put in the form:

(8)

which reduces in the Newtonian case to the classical Hagen-Poiseuille relation .

### Extension of Murray's Law

Let us express Eq. (7) for a vessel *k *included in a tree structure, it comes:

(9)

In a more general manner (also suggested in [2]), Eq. (9) may be written as:



where Ψ_*κ *_is function of the length *l*_k _of the vessel *k *and the properties of blood, *Q*_*κ *_is the mass flow rate of blood and *R*_*κ *_is the radius of the vessel *k*. The parameters *a *and *b *are only function of the fluid properties. From Eq. (8), these parameters can be identified for a power law fluid as:

(10)

Introduce a cost function Φ_k_, as a linear combination of two quantities: the pumping power *Q*_k_·Δ*p*_k _and the energy cost to maintain the blood volume *π*·*l*_k_·*R*^2^_k_, it yields:

(11)

where *A*_k _is a cost factor for pumping and *B*_k _is a sort of maintenance cost of the blood volume. In other words, the metabolic rate of energy required to maintain the volume of blood. The cost function can be written as:

(12)

where *B*_*k*_' = *B*_*k*·_*π*·*l*_*k *_and *A*_*k*_' = *A*_*k*_·Ψ_*k*_.

The derivative of this expression with respect to the radius at constant mass flow rate and channel length gives the value of an extremum:

(13)

The second derivative of the cost function is

(14)

which is found to be always positive because b>0, and so are *A*_k_' and *B*_k_'. As a result, the extremum is a minimum.

According to Eq. (13), the relation between the radius *R*_k _and the mass flow rate *Q*_k _is as follows:

(15)

It is noteworthy that if the constraint is not the energy cost to maintain the blood volume but that of the internal area of the vessel (2 *π*·*l*_k_·*R*_k_), we get:

(16)

This expression may be useful when one wants to include mass and/or heat transfer through the vessel wall. Actually, when the vessel diameter decreases, blood catch up with a non-Newtonian fluid and the heat and mass transfer through the vessel wall becomes more and more significant.

**Figure 2:**
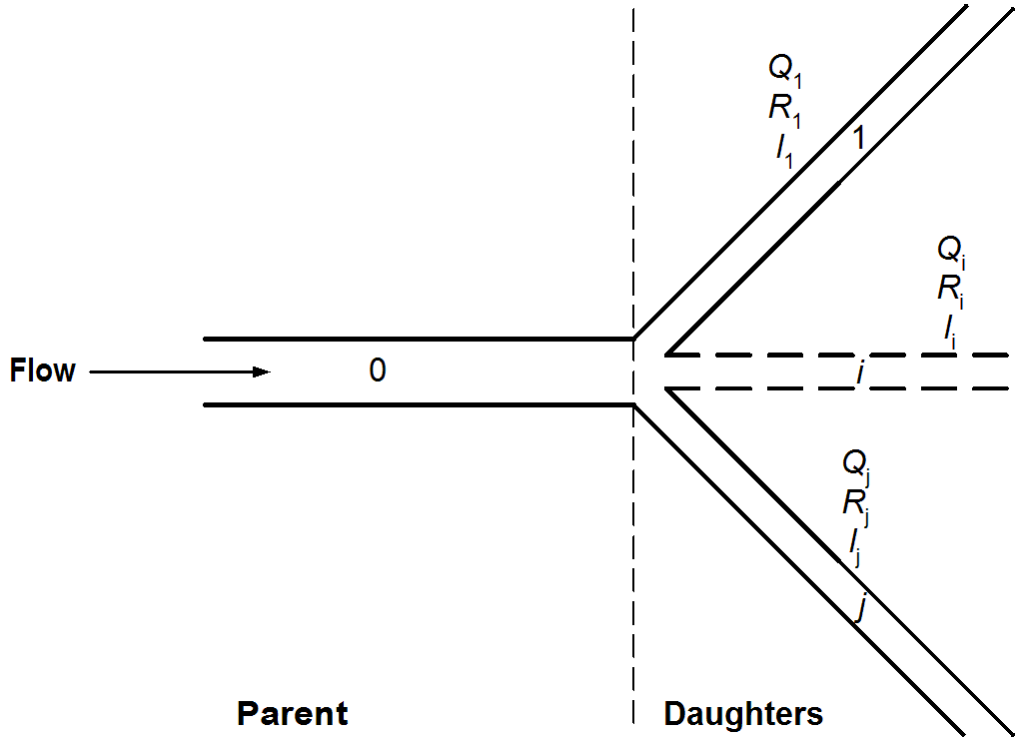
**Schematic of a bifurcation: parent vessel 0 divided into *j *daughter vessels**.

Now consider a parent vessel (0) divided into a finite number of vessels (*1*,..., *j*) as shown in Fig. (2). Conservation of mass yields the following relation:

(17)

Combining Eqs. (17) and (15), we get:

(18)

Equation (18) is the generalization of the cube law. In case of laminar Newtonian flow (*a *= 1 and *b *= 4, thus *c *= 3), the classical cube law is recovered. Note that whatever the value of *n *in the Poiseuille case, *c *is equal to 3.

The ratio of two consecutive vessels (parent and daughter) is thus written as:

(19)

Expression (19) is the generalization of Murray's law and was also proposed by [32]. For instance, if one assumes a Poiseuille flow and two daughter vessels one gets the well known result:



Relation (19) takes into account a general form of the pressure drop (not necessarily a Poiseuille flow), a finite but not fixed number of daughter vessels (but not necessarily equal to two) and a possible unequal distribution of the flow in each daughter vessel. Two interesting parameters may thus be calculated:

- The bifurcation index *α*_i _represents the relative caliber of the symmetry of the bifurcation:

(20)

- The area ratio (expansion parameter) *β *which is the ratio of the combined cross-sectional area of the daughters over that of the parent vessel. Values of *β *greater than unity produce expansion in the total cross-sectional area available to flow as it progresses from one of the tree to the next. It can be written as:

(21)

The following relations are then deduced:

(22)

(23)

Until now, all equations are formulated with a parent vessel that divid into a finite number of daughter vessels (*1*,..., *j*). However, since a parent vessel divide into two daughter vessels in animals and humans, further equations will be formulated with *j *= 2.

### Meaning of the results

In this section, we will examine the meaning of the results obtained in the general case. Particularly, we will focus on the variation of each parameter with the radius of the vessel.

#### Volumetric flow

As established above, see Eq. (15), the volumetric flow rate is proportional to the radius to the power *c *when minimizing the cost function:



#### Velocity of flow

The volumetric flow is proportional to *R*^*c *^and the cross-area of a vessel is proportional to *R*^2^, thus the flow velocity (*v*) is expressed as:



#### Velocity profile

The maximum velocity, denoted by *v*_max_, is attained at the center of the vessel. It can thus be obtained by setting *r *to 0 in Eq. (5).

The mean velocity <*v*> is defined as the ratio between the flow rate (Eq. (6)) and the vessel cross section area:



From these expressions, the velocity profile is independent of the radius of the vessel and is given by the following relation:



As a result, the velocity profile is only function of the fluid properties and remains the same whatever the radius.

#### Vessel wall shear stress

The vessel wall shear stress may be expressed as:



In the particular case of *c *= 3, the vessel wall shear stress remains unchanged all along the vascular system.

If *c*<3, when blood flows from the parent to the daughter vessels, the vessel wall shear stress increases because the vessel radii decrease in the flow direction. On the contrary, if *c*>3, the vessel wall shear stress decreases because the vessel radii increase in the flow direction.

#### Reynolds number

The Reynolds number (Re) is proportional to the radius *R *multiplied by the flow velocity *v*. According to the relation obtained above for the velocity of flow, it comes:



Since *c *is often greater than two, the Reynolds number will always increases in the direction of the blood path.

#### Pressure gradient

The pressure gradient is proportional to *Q*^a^/*R*^b^, i.e. *R*^ca^/*R*^b ^since *Q *∝ *R*^*c*^. The relation between the pressure drop and the vessel radius is then:



#### Conductance and resistance

In a Murray system, Σ*R*^*c *^is constant. In addition, the resistance of the fluid is proportional to *R*^-*b*^. We thus obtain:



and the reciprocal of resistance is the conductance defined as:



#### Cross sectional area

Σ*R*^*c *^is constant in a Murray system and the cross sectional area of vessels is proportional to Σ*R*^-2^. As a consequence, the cross sectional area is:



#### Entropy generation

Entropy generation has several origins: heat transfer, mass transfer, pressure drop... Entropy generation depends on the internal physical phenomena encountered in a process. In the case of an isothermal flow, entropy generation exists, can be quantified, and is related to mass transfer and pressure drop. In our case, entropy generation (*S'*) may be obtained by dividing the cost function (based on the surface constraint) by temperature, which is assumed uniform and constant here (*T *= 310.15K) [2]. The volume constraint cannot be used in that case because the cost of blood maintenance is a process which is external to the system. The minimum entropy generation is thus reached at the minimum of the cost function. In case of surface constraint, the expression of the entropy generation is defined as:

(25)

where *A*_*k*_" = *A*_*k*_·Ψ_*k*_/*T *and *B*_*k*_" = *B*_*k*_·2·*π*·*l*_*k*_/*T*. Combining with Eq. (10), Expression (25) reduces to



In case of two daughter vessels, the link between the entropy generation upward and downward the arterial bifurcation is defined as:

(26)

For the Newtonian case, *c *= 2.5 and  = 1.52. For the non-Newtonian case, *n *= 0.74 for instance and  = 1.50. Whatever the fluid, as  >1, the entropy generated in all the daughter vessels is greater than the entropy generated in the parent vessel. Furthermore, this result means that the difference of entropy generation between the parent and daughter vessels is smaller for a non-Newtonian fluid than for a Newtonian fluid. This behaviour can be related to the velocity profile, which is blunter for a non-Newtonian fluid, as shown by Eq. (5).

### Illustrating example

Egushi and Karino [31] measured blood viscosity as a function of shear rate using the classical cone-plate viscometer and obtained



Thanks to a falling-ball viscometer, they obtained:



Table 1 shows the effect of *n *on the *c *parameter for both constraints. When the cost function involves the volume constraint, parameter *c *equals three whether the fluid is described by the Newtonian law or by a power-law model. In that case, Murray's law remains valid for a shear-thinning fluid like blood. The optimal ratio between a parent vessel radius and daughter radii is the same whether the fluid is Newtonian or not. In contast, parameter *c *depends on the value of *n *when the cost function involves the surface constraint. The optimal ratio of parent vessel radius to daughter radii thus depends on the fluid properties. It is found that decreasing *n *leads to a drop of parameter *c*. In conclusion, the more shear-thinning the fluid is, the lower the optimal ratio. *c *thus ranges from 2.42 to 3, depending on the constraint, which corresponds to the typical range of *c *measured experimentally and reported in the literature [4-8].

**Table 1:** Influence of *n *on the *c *parameter for the two different constraints.

Nominal value of *n*	*n*	Volume constraint	Surface constraint
		*c*	*C*

1	1	3	2.5

0.81	0.78	3	2.44
	
	0.81	3	2.45
	
	0.84	3	2.46

0.74	0.72	3	2.42
	
	0.74	3	2.43
	
	0.76	3	2.43

Table 2 summarizes the influence of index *n *on different parameters. For example, when assuming a volume constraint, the Reynolds number varies as the radius to power two. It means that whatever the fluid model, the Reynolds number follows the same law. For a surface constraint, decreasing *n *leads to a decrease in the exponent of the radius. In other words, a shear-thinning fluid the Reynolds number varies less with the vessel radius, all other parameters being kept constant. In addition, the flow resistance is proportional to *R *to the power *c' *where *c' *is always negative. This result agrees with the fact that the greatest part of the resistance of the arterial tree is in the smallest vessels.

**Table 2:** Influence of *n *on different parameters

Parameters	Newtonian *n *= 1		Non-Newtonian *n *= 0.81		Non-Newtonian *n *= 0.74	
	Σ*R*^3^	Σ*R*^2.5^	Σ*R*^3^	Σ*R*^2.45^	Σ*R *^3^	Σ*R *^2.43^

Volumetric flow	*R*^3^	*R*^2.5^	*R*^3^	*R*^2.45^	*R*^3^	*R*^2.43^

Velocity of flow	*R*	*R*^0.5^	*R*	*R*^0.45^	*R*	*R*^0.43^

Vessel wall shear stress	1	*R*^-0.5^	1	*R *^-0.55^	1	*R *^-0.57^

Reynolds number	*R*^2^	*R*^1.5^	*R*^2^	*R*^1.45^	*R*^2^	*R*^1.43^

Pressure gradient	*R *^-1^	*R *^-1.5^	*R *^-1^	*R*^-1.45^	*R*^-1^	*R *^-1.42^

Conductance	*R*	*R*^1.5^	*R*^0.43^	*R*^0.98^	*R*^0.22^	*R*^0.79^

Resistance	*R*^-1^	*R*^-1.5^	*R*^-0.43^	*R*^-0.98^	*R*^-0.22^	*R*^-0.79^

Cross sectional area	*R*^-1^	*R*^-0.5^	*R*^-1^	*R*^-0.45^	*R*^-1^	*R*^-0.43^

Entropy generation	*R*2	*R*2	*R*^2^	*R*	*R*^2^	*R*

*β*and	1.26	1.52	1.26	1.51	1.26	1.50

## Conclusion

Blood is a multi-component mixture with complex rheological characteristics. Experimental investigations have shown that blood exhibits non-Newtonian properties such as shear-thinning, viscoelasticity, thixotropy and yield stress. Blood rheology is shown to be related to its microscopic structures (e.g. aggregation, deformation and alignment of blood cells and plattelets). Shear-thinning is the predominant non-Newtonian effect in bifurcations of blood flows.

In this study, we have proposed for the first time an analytical expression of Murray's law using a non-Newtonian blood flow model (power law model), assuming two different constraints in addition to the pumping power: (i) the volume constraint and (ii) the surface constraint. Surface constraint may be useful if one wants to include heat and/or mass transfer in the cost function, specially in capillaries. For a seek of generality, the relationships have been given for an arbitrary number of daughter vessels. Note that there is an alternative formulation of the constrained optimization problem using the Lagrange multipliers, as discussed in [32]. However, using this approach, the results presented in this paper would not have been modified.

It has been showed that for a cost function including the volume constraint, classical Murray's law remains valid (i.e. Σ*R*^*c *^= *cste *with *c *= 3 is verified). In other words, the value of *c *is independent of the fluid properties. On the contrary, for a cost function including the surface constraint, different values of *c *may be calculated depending on the fluid properties, i.e. the value of *n*. The fluid is shear-thinning if *n*<1 and shear-thickening if *n*>1. When *n *= 1 the Newtonian fluid is recovered. In the present study, we have used two different blood values of *n *found in the literature, namely *n *= 0.81 and *n *= 0.74. In summary, it has been found that *c *varies from 2.42 to 3 depending on the constraint and the index *n*. For the particular Newtonian model, the surface constraint leads to *c *= 2.5.

Entropy generation has several origins: heat transfer, mass transfer, pressure drop, etc. The cost function (based on the surface constraint) can be related to entropy generation by dividing it by the temperature. It has been demonstrated that the entropy generated in all the daughter vessels is greater than the entropy generated in the parent vessel. Furthermore, it is shown that the difference of entropy generation between the parent and daughter vessels is smaller for a non-newtonian fluid than for a Newtonian fluid. This behaviour can be related to the velocity profile, which is blunter for a non-Newtonian fluid, as shown by Eq. (5).

Based on the literature review and on our work, we propose in the following, further possible investigations:

- The effect of singularities on the cost function has hardly ever been investigated. Few works exist on this aspect [33] and Tondeur et al. (Tondeur D, Fan Y, Luo L: Constructal optimization of arborescent structures with flow singularities. Chem. Eng. Sci. 2009, submitted.). However, the effect of the singularities on the entropy generation might not be negligible. This contribution should be added in the cost function in the future.

- In reality, heat and mass might be transfered through the vessel wall, leading to resistance that should be included in the cost function. Indeed, gases, nutrients and metabolic waste products are exchanged between blood and the underlying tissue. Substances pass through the vessels by active or passive transfer, i.e. diffusion, filtration or osmosis. Moreover, pathologic states such as edema and inflammation might increase such phenomena.

- It is accepted that pulsatile blood flow is more realistic than steady-state flow. The cost function should also include the effect of pulsatile flow in an elastic tube.

- Blood is essentially a two-phase fluid consisting of formed cellular elements suspended in a liquid medium, the plasma. The corpuscular nature of blood raises the question of whether it can be treated as a continuum, and the peculiar makeup of plasma makes it seem different from more common fluids. In particular, when the vessel radius decreases down to the smallest capillaries, the continuum approach diverges from the reality. Treating blood as a non-continuum fluid should be a possible next step.

## Competing interests

The authors declare that they have no competing interests.

## Authors' contributions

RR developed the general equations of Murray's law and carried out the entropy generation analysis.

FR developed the equations for the non-Newtonian model of blood flow.

DB participated in the design of the study and the manuscript.

JB participated in the design of the study and the manuscript.

All the authors read and approved the final manuscript.
